# Supplementary material for: Identification of a Putative SARS-CoV-2 Main Protease Inhibitor through In Silico Screening of Self-Designed Molecular Library
Source: Int J Mol Sci. 2023 Jul 13;24(14):11390. doi: 10.3390/ijms241411390 (PMC10379331; doi:10.3390/ijms241411390)
Supplement: Supplementary file 1 [file ijms-24-11390-s001.zip › ijms-2441435-supplementary.pdf]

Article

Identification of a Putative SARS-COV-2 Main Protease Inhibitor through In Silico Screening of Self-designed Molecular Library

Nanxin Liu [1], Zeyu Yang [1], Yuying Liu [1], Xintao Dang [1], Qingqing Zhang [1], Jin Wang [1], Xueying Liu [2], Jie Zhang [1] and Xiaoyan Pan [1,*]

[1] School of Pharmacy, Health Science Center, Xi'an Jiaotong University, Xi’an, 710061, China.
[2] School of Pharmacy, The Fourth Military Medical University, Xi'an, 710032, China.
* Correspondence: panxy2016@xjtu.edu.cn.

Table S1. Docking Score of All Compounds

Title	SMILE	docking binding energy(ΔG, kcal/mol)
V1	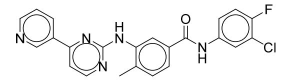	-6.626
V2	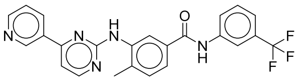	-6.472
V3	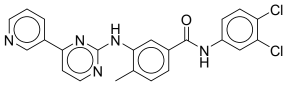	-6.52
V4	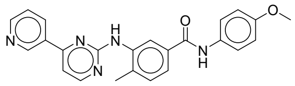	-6.527
V5	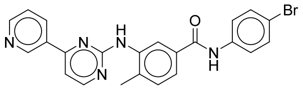	-6.36
V6	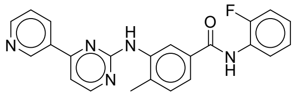	-6.86
V7	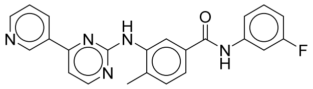	-6.711
V8	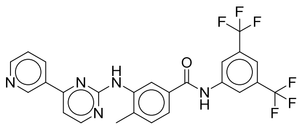	-6.221
V9	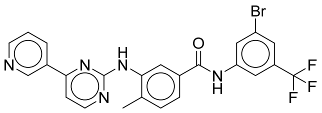	-6.475
V10	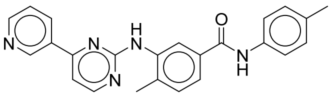	-6.533
V11	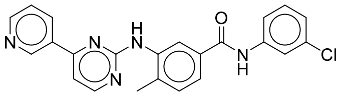	-6.466
V12	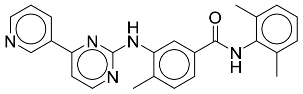	-7.192
V13	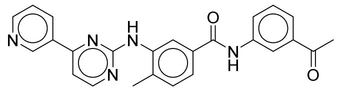	-6.733
V14	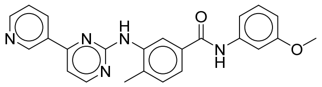	-6.682

V15	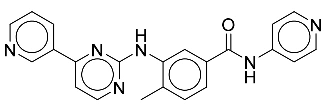	-6.136
V16	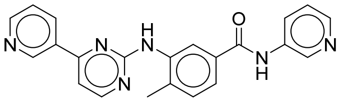	-6.321
V17	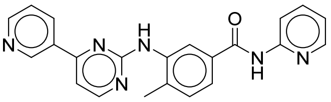	-6.407
V18	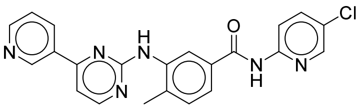	-6.411
V19	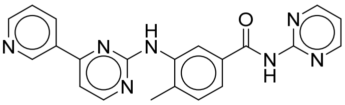	-6.101
V20	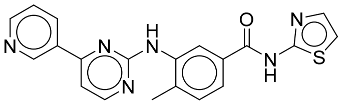	-6.529
V21	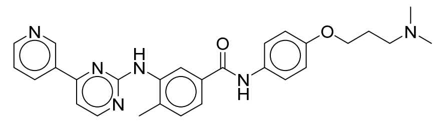	-6.763
V22	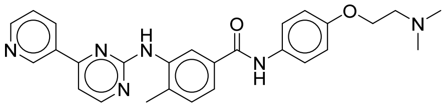	-6.061
V23	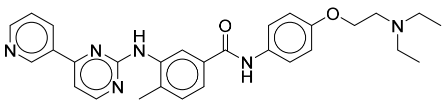	-6.659
V24	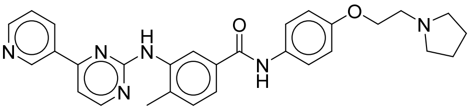	-5.908
V25	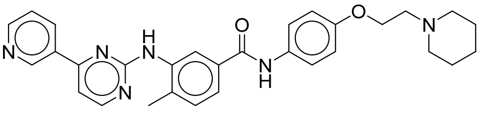	-5.956
V26	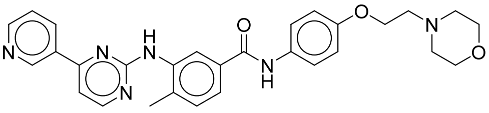	-5.842
V27	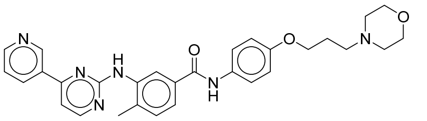	-6.12
V28	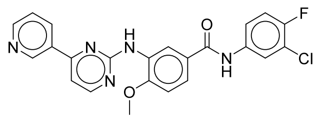	-5.487

V29	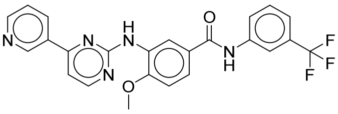	-6.303
V30	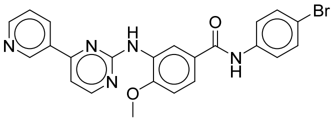	-6.415
V31	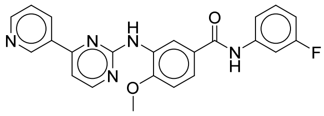	-6.799
V32	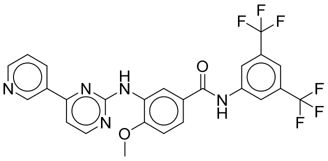	-5.602
V33	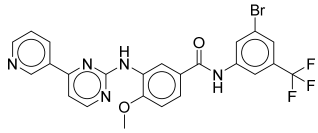	-6.088
V34	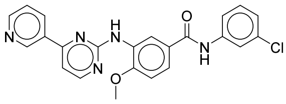	-6.715
V35	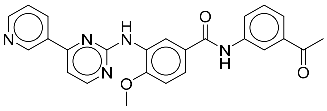	-6.282
V36	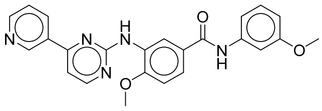	-6.126
V37	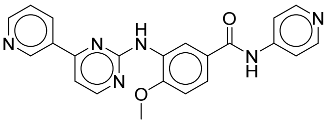	-6.401
V38	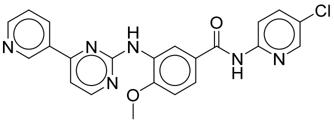	-5.925
V39	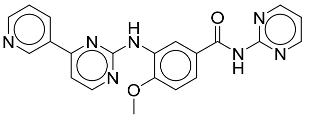	-6.62
V40	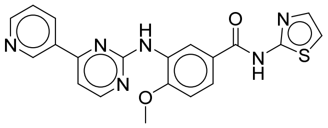	-6.268
V41	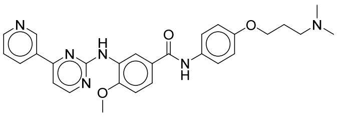	-5.188
V42	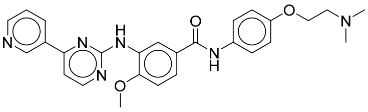	-5.813

V43	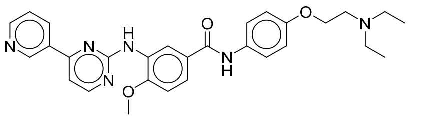	-6.381
V44	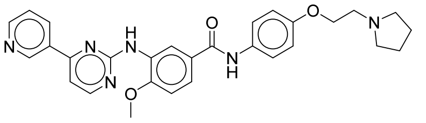	-6.525
V45	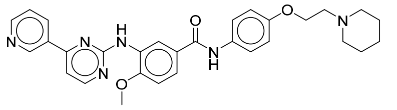	-6.403
V46	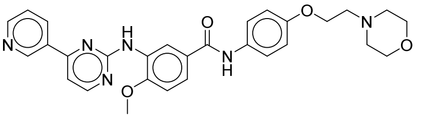	-5.05
V47	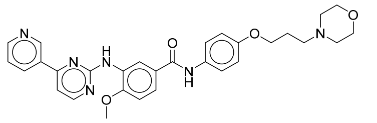	-6.01
V48	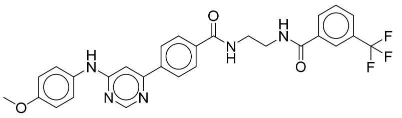	-5.521
V49	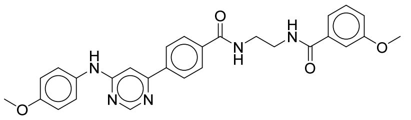	-6.328
V50	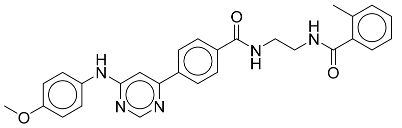	-5.535
V51	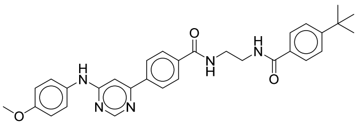	-6.039
V52	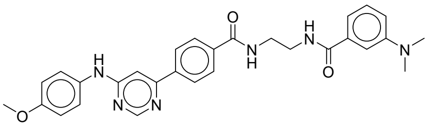	-5.586
V53	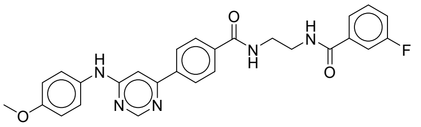	-6.355
V54	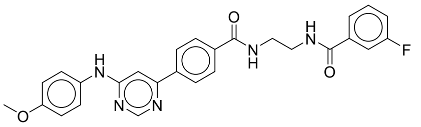	-6.813
V55	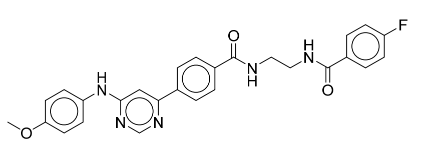	-6.767
V56	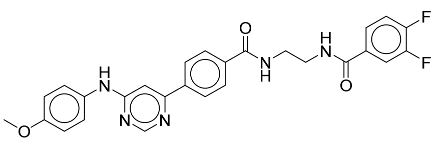	-6.645

V57	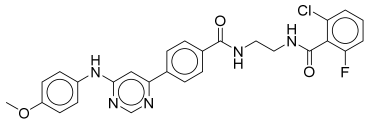	-5.978
V58	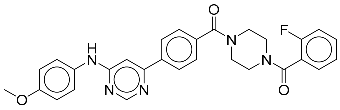	-7.013
V59	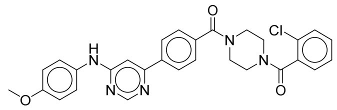	-6.951
V60	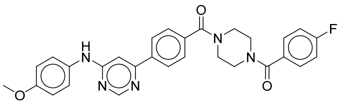	-7.261
V61	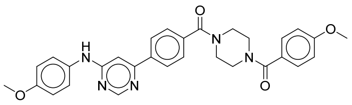	-6.346
V62	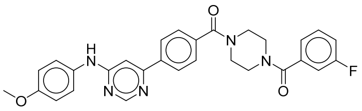	-6.572
V63	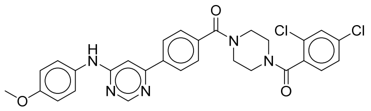	-6.43
V64	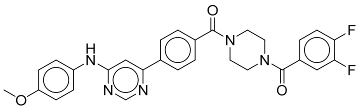	-6.551
V65	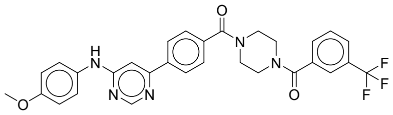	-6.611
V66	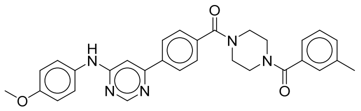	-6.883
V67	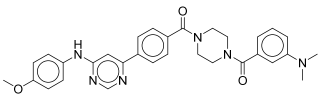	-6.629
V68	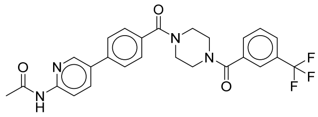	-5.99
V69	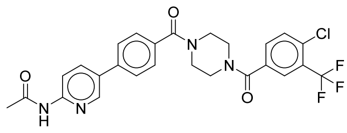	-5.648
V70	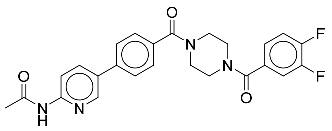	-7.299

V71	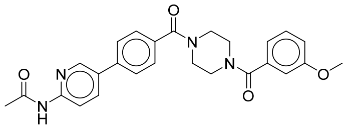	-6.066
V72	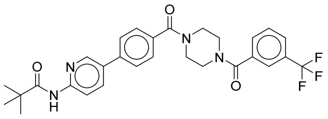	-6.833
V73	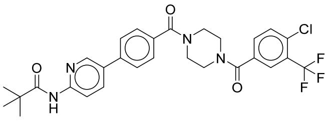	-6.759
V74	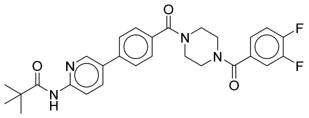	-7.225
V75	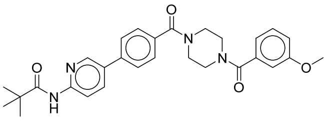	-7.331
V76	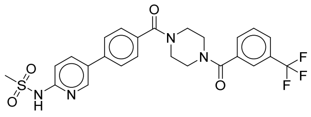	-6.335
V77	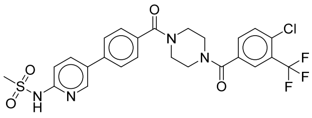	-5.902
V78	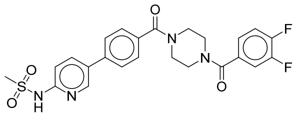	-6.866
V79	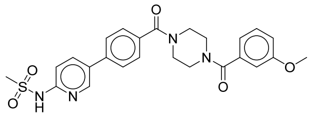	-6.627
V80	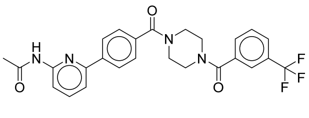	-6.066
V81	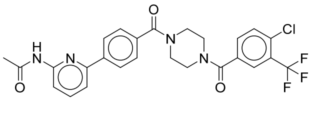	-6.061
V82	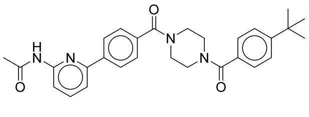	-5.21
V83	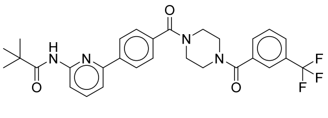	-6.68
V84	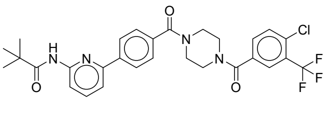	-6.903

V85	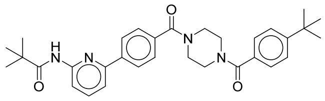	-5.975
V86	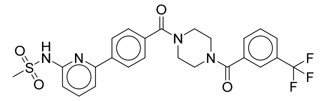	-7.085
V87	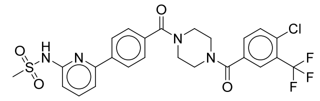	-6.971
V88	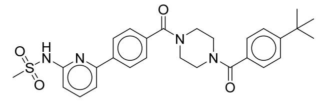	-4.961
V89	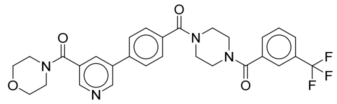	-6.948
V90	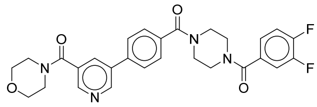	-6.195
V91	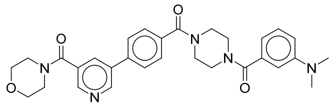	-6.571
V92	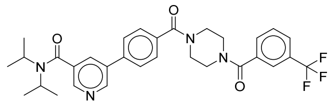	-6.089
V93	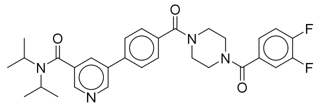	-5.944
V94	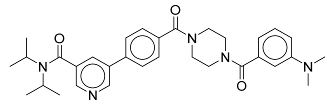	-6.336
V95	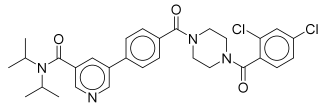	-6.179
V96	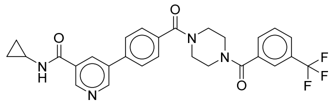	-6.921
V97	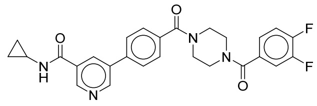	-7.138
V98	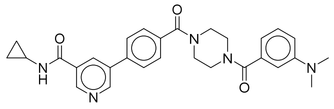	-6.929

V99	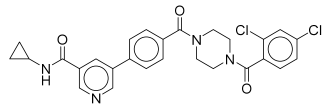	-6.541
V100	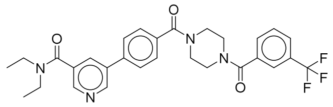	-6.713
V101	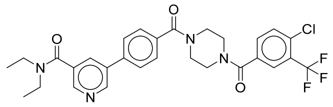	-5.911
V102	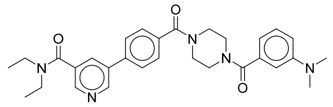	-5.655
V103	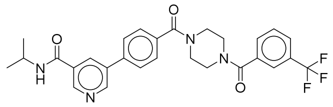	-7.535
V104	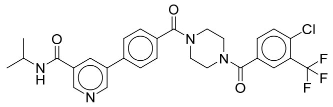	-6.66
V105	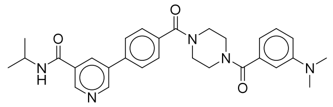	-6.812
V106	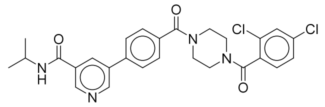	-6.927
V107	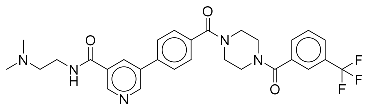	-6.807
V108	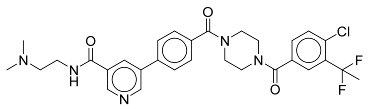	-5.975
V109	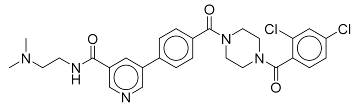	-7.567
V110	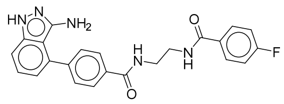	-6.331
V111	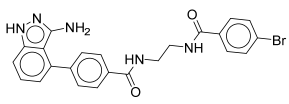	-7.195
V112	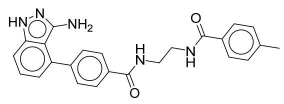	-7.29

V113	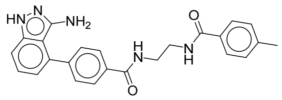	-5.688
V114	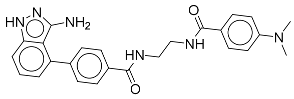	-6.728
V115	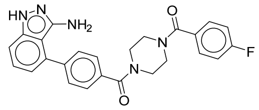	-6.622
V116	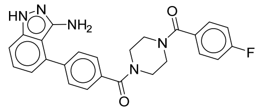	-6.331
V117	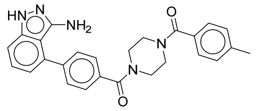	-5.895
V118	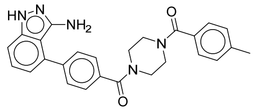	-6.412
V119	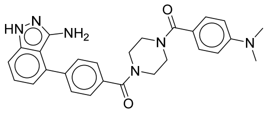	-5.765
V120	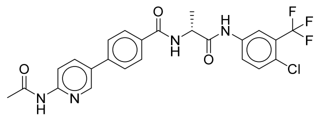	-7.081
V121	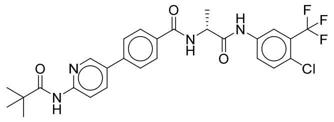	-5.925
V122	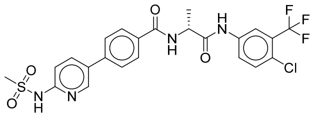	-7.095
V123	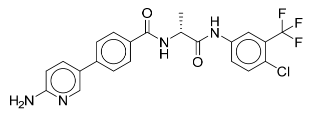	-6.219
V124	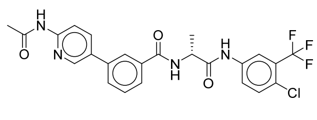	-6.607
V125	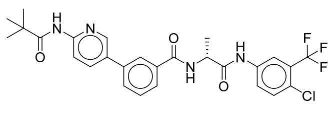	-6.991
V126	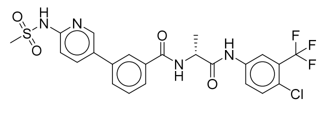	-6.89

V127	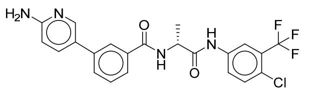	-6.773
V128	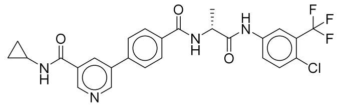	-7.264
V129	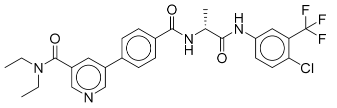	-6.636
V130	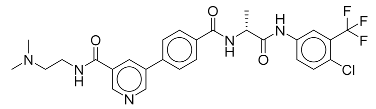	-6.44
V131	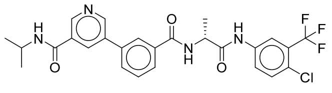	-7.876
V132	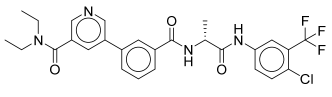	-6.415
V133	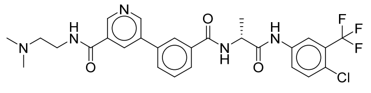	-8.379
V134	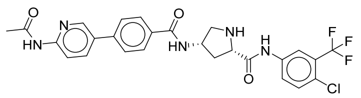	-5.713
V135	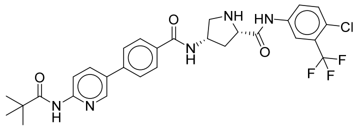	-6.087
V136	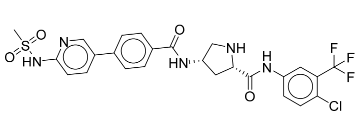	-6.662
V137	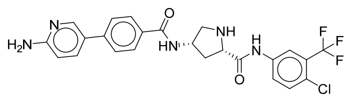	-6.378
V138	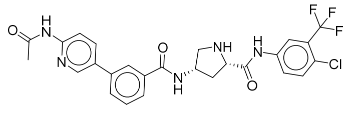	-6.75
V139	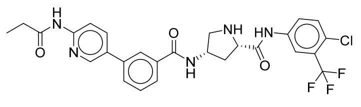	-7.422
V140	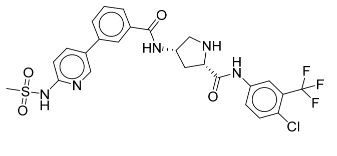	-6.773

V141	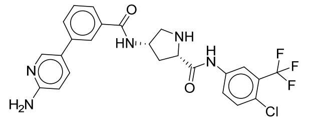	-6.33
V142	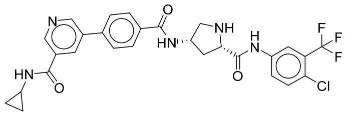	-6.301
V143	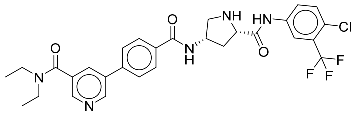	-6.433
V144	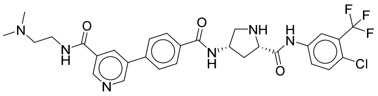	-7.195
V145	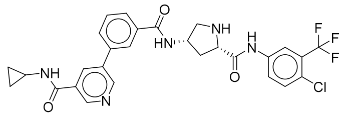	-6.482
V146	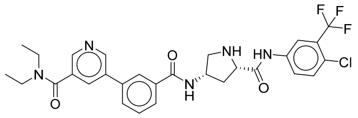	-6.017
V147	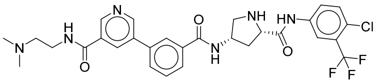	-7.364
V148	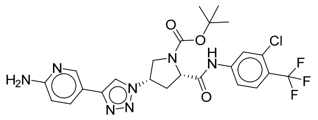	-6.25
V149	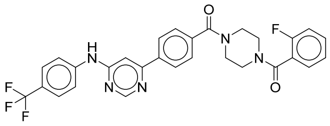	-6.896
V150	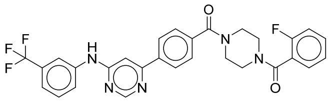	-7.145
V151	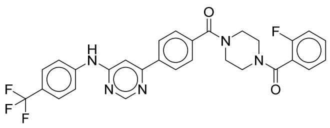	-6.441
V152	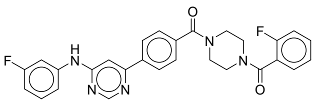	-6.778
V153	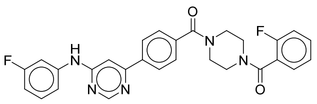	-6.788
V154	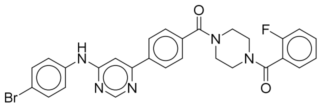	-7.26
V155	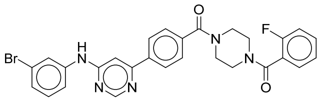	-7.113

V156	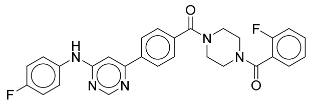	-6.984
V157	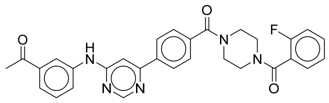	-6.881
V158	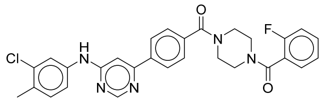	-6.892
V159	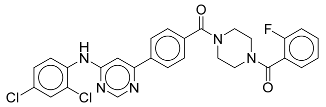	-7.091
V160	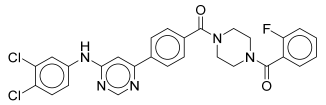	
V161	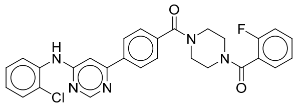	-6.514
V162	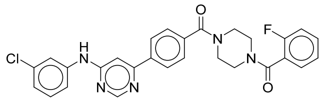	-6.545
V163	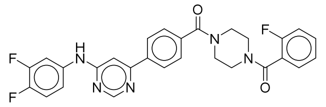	-7.052
V164	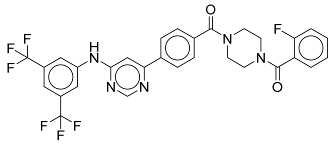	-6.587
V165	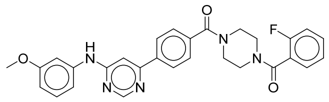	-7.051
V166	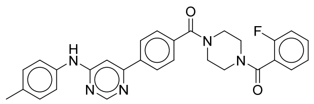	-6.978
V167	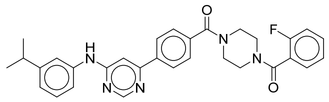	-6.702
V168	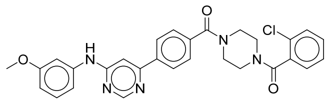	-7.182
V169	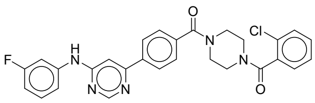	-6.611

V170	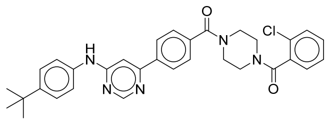	-7.085
V171	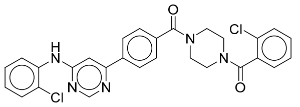	-6.902
V172	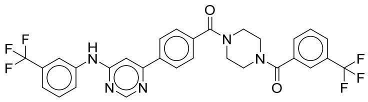	-7.403
V173	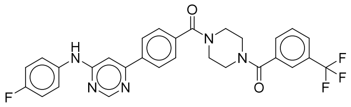	-7.138
V174	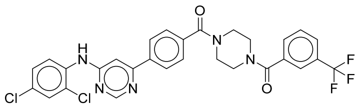	-7.369
V175	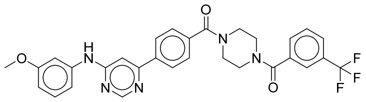	-7.538
V176	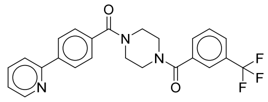	-6.275
V177	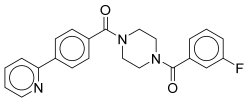	-6.861
V178	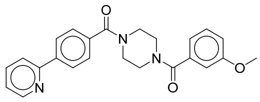	-6.171
V179	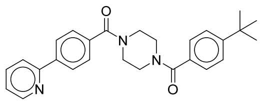	-5.036
V180	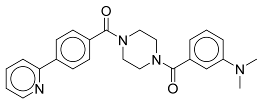	-6.406
V181	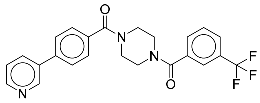	-5.335
V182	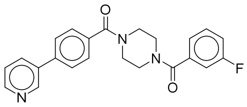	-5.516
V183	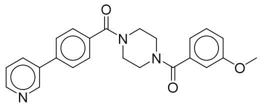	-6.283

V184	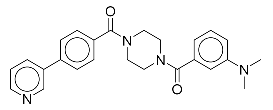	-5.509
V185	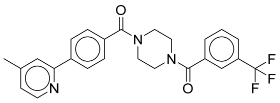	-6.401
V186	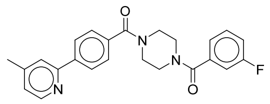	-6.067
V187	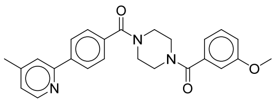	-6.328
V188	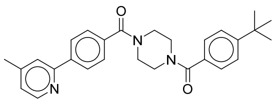	-5.094
V189	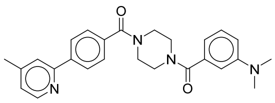	-6.027
V190	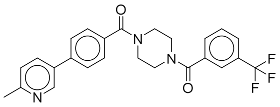	-6.42
V191	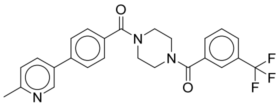	-5.786
V192	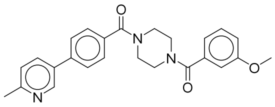	-6.034
V193	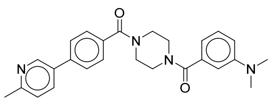	-5.887
V194	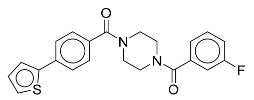	-6.209
V195	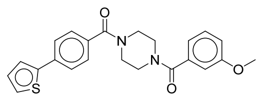	-5.696
V196	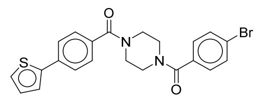	-5.185
V197	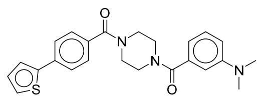	-5.434

V198	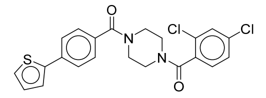	-6.823
V199	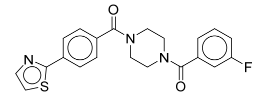	-5.635
V200	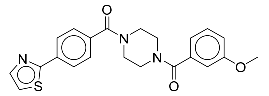	-5.655
V201	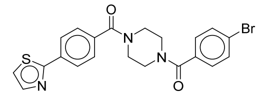	-5.182
V202	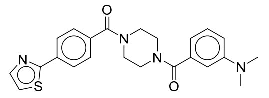	-6.075
V203	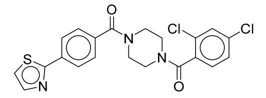	-6.661
V204	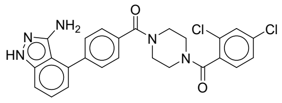	-7.89
V205	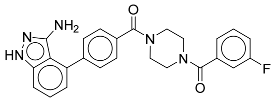	-7.501
V206	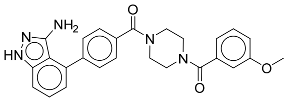	-6.213
V207	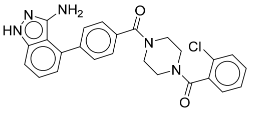	-6.222
V208	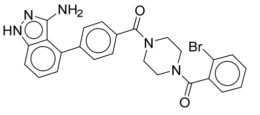	-6.951
V209	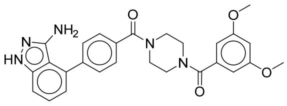	-6.811
V210	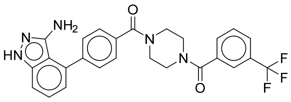	-6.676
V211	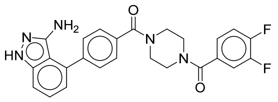	-6.714

V212	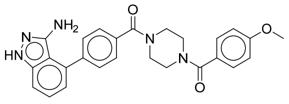	-7.688
V213	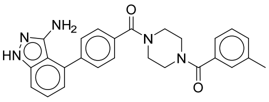	-6.946
V214	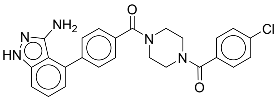	-5.958
V215	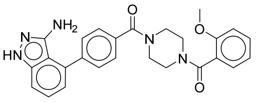	-7.032
V216	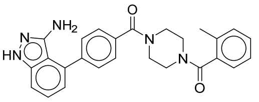	-6.946
V217	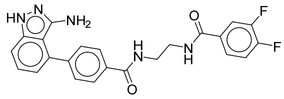	-6.482
V218	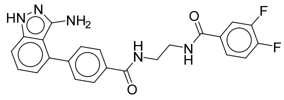	-5.841
V219	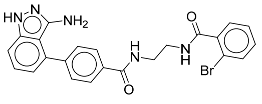	-7.212
V220	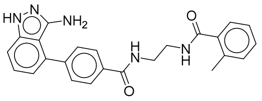	-6.013
V221	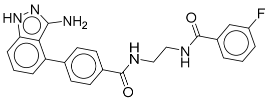	-6.943
V222	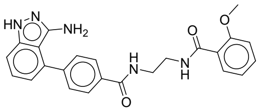	-7.305
V223	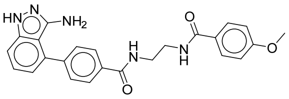	-6.69
V224	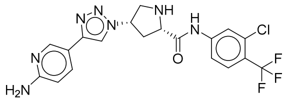	-6.764
V225	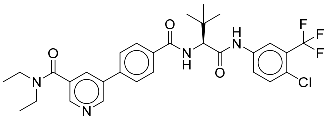	-7.068

V226	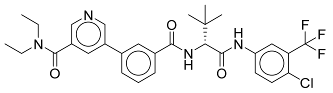	-7.141
V227	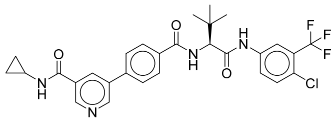	-6.403
V228	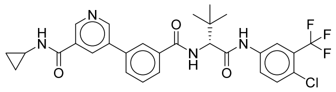	-8.122
V229	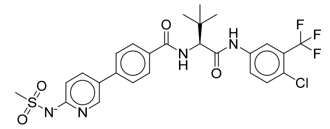	-7.509
V230	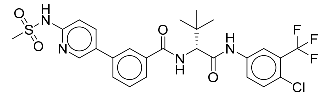	-7.314
V231	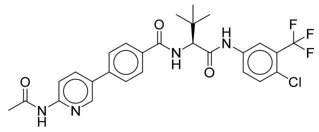	-7.372
V232	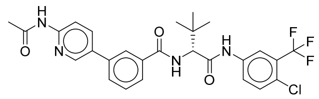	-6.088
V233	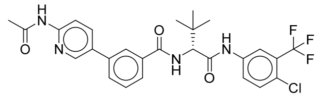	-6.277
V234	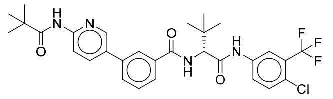	-6.263
V235	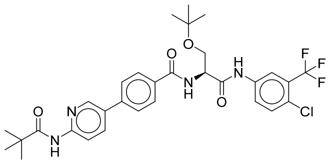	-6.649
V236	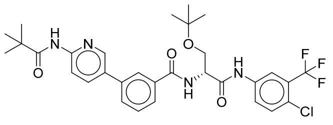	-6.902
V237	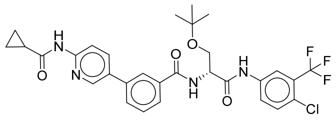	-6.847
V238	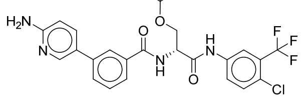	-7.134

V239	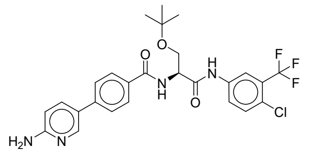	-6.542
V240	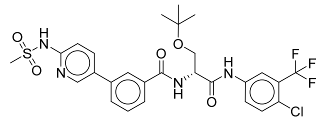	-7.211
V241	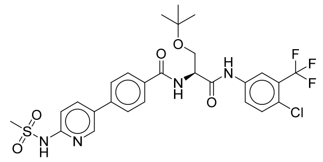	-7.108
V242	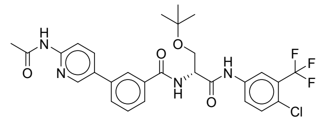	-6.218
V243	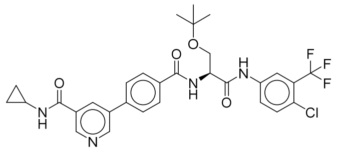	-7.68
V244	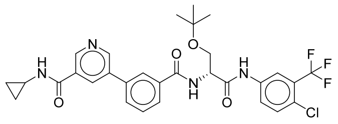	-6.494
V245	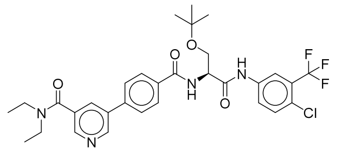	-7.401
V246	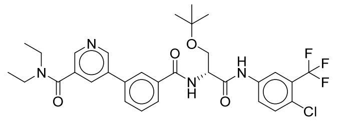	-6.602
V247	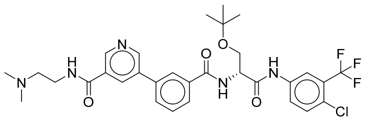	-8.538
V248	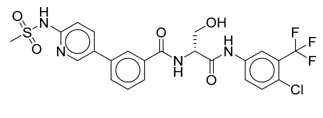	-7.565
V249	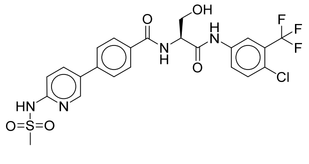	-6.568
V250	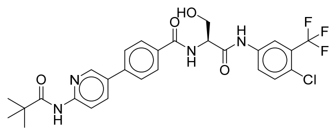	-6.449

V251	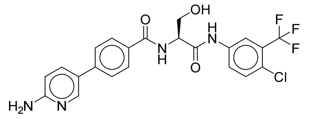	-5.414
V252	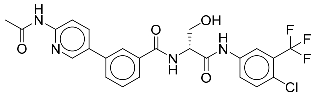	-7.416
V253	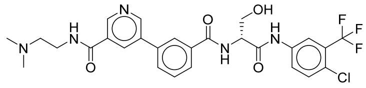	-8.832
V254	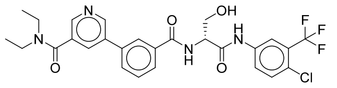	-7.286
V255	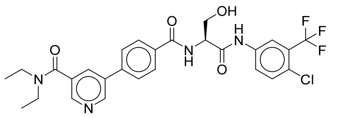	-5.04
V256	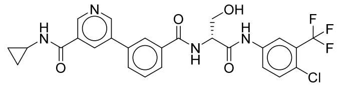	-6.19
V257	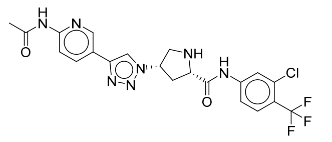	-7.37
V258	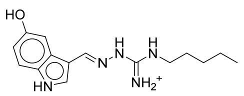	-3.553
V259	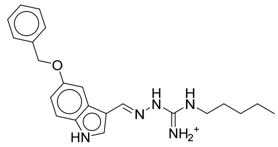	-5.516
V260	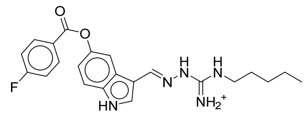	-6.287
V261	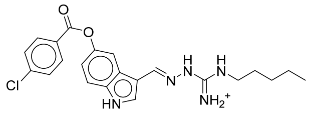	-6.367
V262	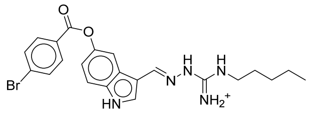	-5.971
V263	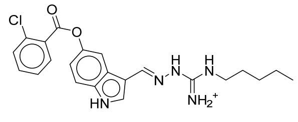	-4.862
V264	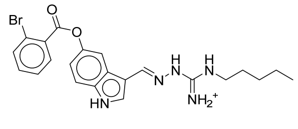	-5.369

V265	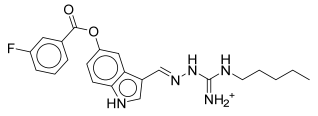	-5.701
V266	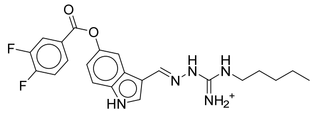	-5.966
V267	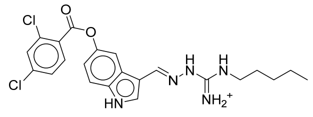	-5.793
V268	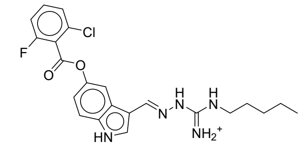	-5.227
V269	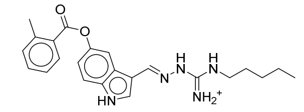	-5.781
V270	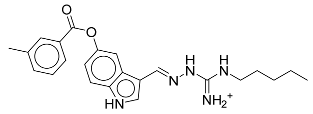	-5.137
V271	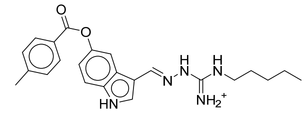	-6.034
V272	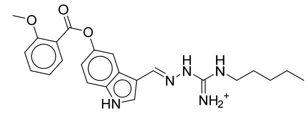	-6.007
V273	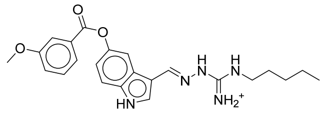	-6.396
V274	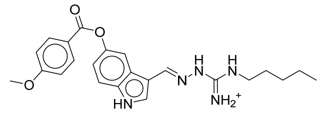	-5.773
V275	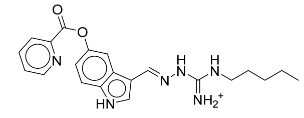	-5.466
V276	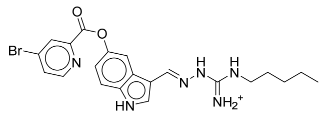	-6.569
V277	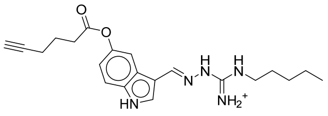	-4.427
V278	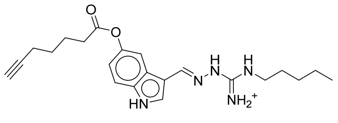	-5.387

V279	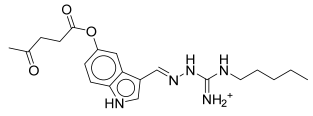	-4.871
V280	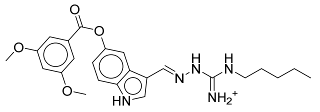	-4.957
V281	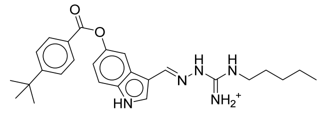	-5.4
V282	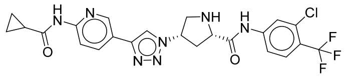	-7.481
V283	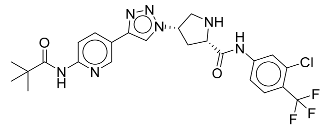	-7.532
V284	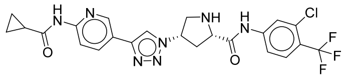	-6.727
V285	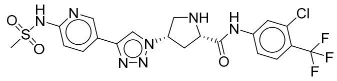	-6.488
V286	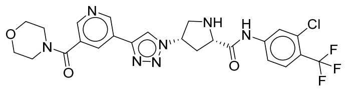	-7.432
V287	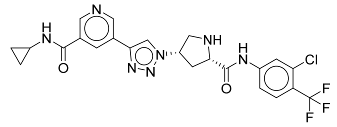	-6.09
V288	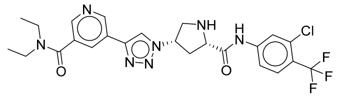	-6.442
V289	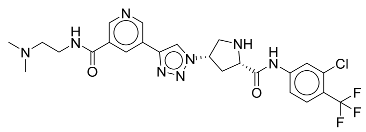	-6.649
V290	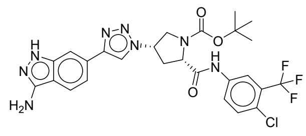	-6.502
V291	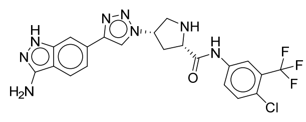	-8.071
V292	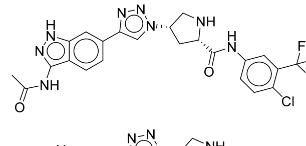	-6.936
V293	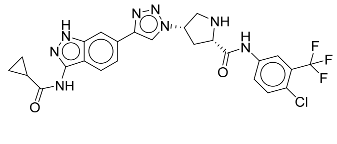	-7.205

V294	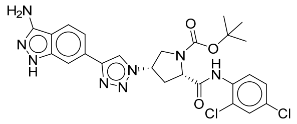	-6.207
V295	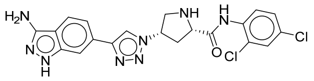	-6.849
V296	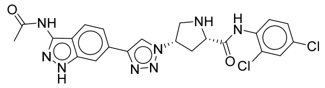	-5.938
V297	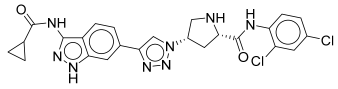	-6.48
V298	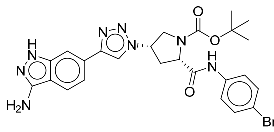	-6.864
V299	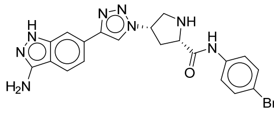	-6.747
V300	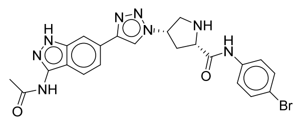	-6.622
V301	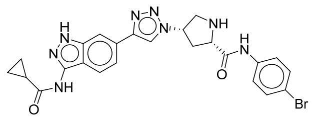	-6.43
V302	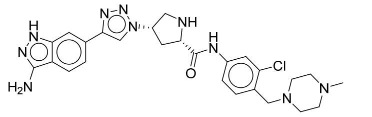	-5.789
V303	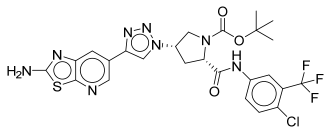	-7.458
V304	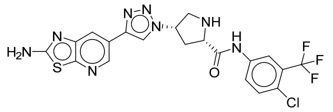	-7.878
V305	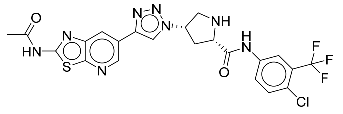	-6.156
V306	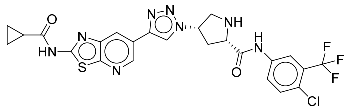	-7.335
V307	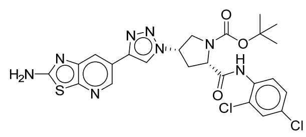	-6.666

V308	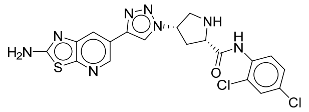	-6.237
V309	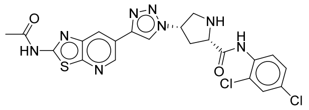	-6.923
V310	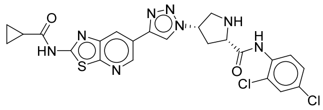	-6.808
V311	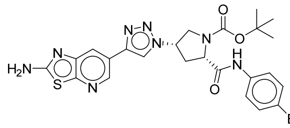	-6.514
V312	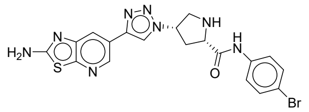	-6.785
V313	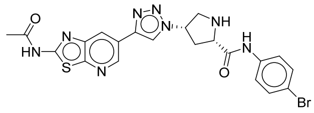	-6.472
V314	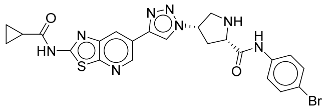	-6.415
V315	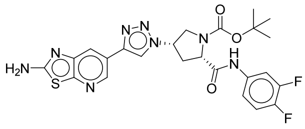	-6.398
V316	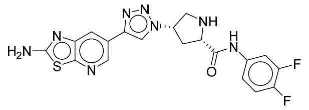	-6.937
V317	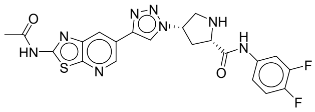	-6.556
V318	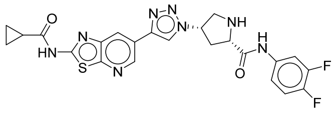	-5.716
Nilotinib	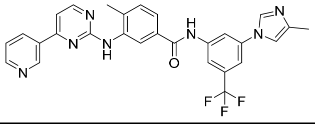	-7.026

Table S2. The interaction between molecule and M^{pro} (based on the IFD docking results)

Compound	Molecular Interactions	Nature of Interactions	Distance (Å)
V247	Lig:N1 - Glu166:OE2	Salt bridge	3.81
	Asn142:HD21 - Lig:O4	Hydrogen Bond	2.10
	Lig:H15 - His164:O	Hydrogen Bond	3.26
	Lig:H11 - Glu166:O	Hydrogen Bond	1.96
	Lig:H36 - Glu166:O	Hydrogen Bond	2.69
	Gln189:HE21 - Lig:O1	Hydrogen Bond	1.96
	Gln192:HE21 - Lig:N3	Hydrogen Bond	2.60
V253	Lig:N1 - Glu166:OE2	Salt bridge	3.17
	Lig:H19 - Leu141:O	Hydrogen Bond	1.97
	Gly143:H - Lig:O4	Hydrogen Bond	2.15
	Ser144:H - Lig:O4	Hydrogen Bond	2.68
	Cys145:H - Lig:O4	Hydrogen Bond	2.17
	Lig:H15 - His164:O	Hydrogen Bond	1.78
	Lig:H28 - Glu166:OE2	Hydrogen Bond	2.43
	Lig:H11 - Glu166:O	Hydrogen Bond	2.62
	Gln189:HE21 - Lig:O2	Hydrogen Bond	2.91
	Gln192:H – Lig:N3	Hydrogen Bond	3.17
	Gln192:HE21 – Lig:N3	Hydrogen Bond	2.73
	His41 - Lig	Hydrophobic (Pi-Pi Stacking)	5.47
	His41 - Lig	Hydrophobic (Pi-Pi Stacking)	5.43
V133	Lig:N3 - Glu166:OE2	Salt bridge	3.23
	Gly143:H - Lig:O2	Hydrogen Bond	1.91
	Cys145:H – Lig:O2	Hydrogen Bond	3.33
	Lig:H28 - Glu166:OE2	Hydrogen Bond	2.36
	Lig:H24 – Glu166:O	Hydrogen Bond	2.00
	Lig:H5 - Arg188:O	Hydrogen Bond	2.71
	Gln189:HE21 - Lig:O1	Hydrogen Bond	1.98
V109	Lig:N1 – Glu166:OE2	Salt bridge	2.77
	His41:HD1 – Lig:O2	Hydrogen Bond	2.21
	Asn142:HD21 – Lig:O3	Hydrogen Bond	2.13
	Gly143:H – Lig:O3	Hydrogen Bond	2.12
	Lig:H11 – Glu166:O	Hydrogen Bond	2.30
	Lig:H30 – Glu166:OE2	Hydrogen Bond	1.82
	Gln192:H – Lig:N3	Hydrogen Bond	2.04
	His41 - Lig	Hydrophobic (Pi-Pi Stacking)	5.29
V131	Lig:H19 – His41:NE2	Hydrogen Bond	3.19
	Gly143:H – Lig:O3	Hydrogen Bond	1.82
	Ser144:H – Lig:O3	Hydrogen Bond	2.49
	Cys145:H – Lig:O3	Hydrogen Bond	2.65
	Lig:H14 – His164:O	Hydrogen Bond	1.86
	Lig:H8 – Glu166:O	Hydrogen Bond	2.09
	Gln192:HE21 – Lig:N2	Hydrogen Bond	3.14
	Gln192:H – Lig:N2	Hydrogen Bond	3.36
	Thr26:H – Lig:Cl1	Halogen Bond	2.40
V254	Gly143:H – Lig:O3	Hydrogen Bond	2.23
	Ser144:H – Lig:O3	Hydrogen Bond	2.29
	Cys145:H – Lig:O3	Hydrogen Bond	1.98
	Lig:H18 – His163:NE	Hydrogen Bond	2.02
	Gln182:HE21 – Lig:O1	Hydrogen Bond	1.99
	Thr190:H – Lig:N2	Hydrogen Bond	2.87
	Thr26:H – Lig:Cl1	Halogen Bond	2.65
V282	Lig:H22 – Met49:O	Hydrogen Bond	3.16

	Lig:H14 – Phe140:O	Hydrogen Bond	2.27
	Asn142:HD22 – Lig:O1	Hydrogen Bond	2.42
	Lig:H11 – Asp187:O	Hydrogen Bond	3.31
	Gln189:HE21 – Lig:O2	Hydrogen Bond	1.77
	Gln192:H – Lig:Cl1	Halogen Bond	2.27
V139	Cys44:HG – Lig:O3	Hydrogen Bond	2.97
	Lig:H12 – His164:O	Hydrogen Bond	2.35
	Gln189:HE21 – Lig:O2	Hydrogen Bond	2.08
	Thr26:O – Lig:Cl1	Halogen Bond	3.32
	Thr26:H – Lig:Cl1	Halogen Bond	3.48
V231	Lig:H23 – His164:O	Hydrogen Bond	3.36
	Glu166:H – Lig:O1	Hydrogen Bond	2.59
	Gln189:HE21 – Lig:O3	Hydrogen Bond	1.87
	Lig:H7 – Gln192:O	Hydrogen Bond	2.06
V128	Thr26:H – Lig:O2	Hydrogen Bond	2.14
	Gly143:H- Lig:N3	Hydrogen Bond	2.06
	Lig:H19 – Glu166:O	Hydrogen Bond	1.60
	Glu166:H – Lig:O1	Hydrogen Bond	1.87
	Lig:H5 – Gln189:OE1	Hydrogen Bond	1.87
V160	Thr26:H – Lig:O2	Hydrogen Bond	2.23
	Gly143:H – Lig:O2	Hydrogen Bond	1.85
	Cys145:H – Lig:O1	Hydrogen Bond	3.14
	Lig:H4 – Glu166:O	Hydrogen Bond	2.10
V163	Thr26:H – Lig:O2	Hydrogen Bond	2.27
	Gly143:H – Lig:O1	Hydrogen Bond	1.79
	Cys145:H – Lig:O1	Hydrogen Bond	3.02
V174	Thr26:H – Lig:O2	Hydrogen Bond	2.89
	His41:HD1 – Lig:N3	Hydrogen Bond	2.13
	Gly143:H – Lig:O1	Hydrogen Bond	1.99
	Cys145:H – Lig:O1	Hydrogen Bond	3.12
	Gln192:H – Lig:Cl2	Halogen Bond	1.98
	Gln192:O – Lig:Cl2	Halogen Bond	3.37
	His41 - Lig	Hydrophobic (Pi-Pi Stacking)	4.11
V243	Asn142:HD21 - Lig:O3	Hydrogen Bond	1.91
	Gly143:H - Lig:O4	Hydrogen Bond	2.14
	Glu166:H - Lig:O1	Hydrogen Bond	2.14
	Lig:H1 - Arg188:O	Hydrogen Bond	2.31
	Lig:H24 - Thr26:O	Hydrogen Bond	2.02
	His41 - Lig	Hydrophobic (Pi-Pi Stacking)	4.06
V229	Lig:H23 – His:164	Hydrogen Bond	3.18
	Glu166:H – Lig:O1	Hydrogen Bond	2.92
	Gln189:HE21 – Lig:O4	Hydrogen Bond	1.74
	Gln192:H – Lig:N2	Hydrogen Bond	2.41
V204	Thr26:H – Lig:O2	Hydrogen Bond	1.91
	Gly143:H – Lig:O1	Hydrogen Bond	1.92
	Cys145:H – Lig:O1	Hydrogen Bond	2.66
	His164:O – Lig:H2	Hydrogen Bond	2.39
	Lig:H3 – Arg188:O	Hydrogen Bond	2.03
	Thr24:HG1 – Lig:Cl1	Halogen Bond	3.31
	Thr45:H – Lig:Cl2	Halogen Bond	2.22
	Thr45:OG1 – Lig:Cl2	Halogen Bond	3.09
V291	Lig:N2 - Glu166:OE2	Salt bridge	4.88
	Lig:H14 - Thr26:O	Hydrogen Bond	1.94
	Thr26:H – Lig:N7	Hydrogen Bond	2.92
	Lig:H12 - His41:O	Hydrogen Bond	2.24
	Lig:H19 - Asn142:OD1	Hydrogen Bond	2.21

	Asn142:H - Lig:O1	Hydrogen Bond	2.62
	Gly143:H - Lig:N5	Hydrogen Bond	2.13
	Cys145:H – Lig:N5	Hydrogen Bond	3.00
	Lig:H2 - Glu166:OE1	Hydrogen Bond	1.89
	Lig:H2 - Glu166:OE2	Hydrogen Bond	3.07
	His41 – Lig	Hydrophobic (Pi-Pi Stacking)	4.40
	His41 – Lig	Hydrophobic (Pi-Pi Stacking)	4.58
	His163 - Lig	Hydrophobic (Pi-Pi Stacking)	5.26
V228	Lig:H1 - His41:NE2	Hydrogen Bond	2.63
	Asn142:H - Lig:O2	Hydrogen Bond	2.57
	Lig:H10 - Asn142:OD1	Hydrogen Bond	1.98
	Lig:H1 - His164:O	Hydrogen Bond	3.10
	Lig:H21 - Glu166:OE1	Hydrogen Bond	1.68
	His172:HE2 – Lig:O3	Hydrogen Bond	3.14
	Gln189:HE21 - Lig:O1	Hydrogen Bond	1.91
V170	Thr26:H – Lig:O2	Hydrogen Bond	1.94
	Gly143:H – Lig:O1	Hydrogen Bond	1.97
	Cys145:H – Lig:O1	Hydrogen Bond	2.88
	Lig:H12 – Glu166:O	Hydrogen Bond	2.32
	Thr24:HG1 – Lig:Cl1	Hydrogen Bond	3.21
	His41 - Lig	Hydrophobic (Pi-Pi Stacking)	5.36
V75	Thr26:H – Lig:O2	Hydrogen Bond	1,99
	Gly143:H – Lig:O3	Hydrogen Bond	1.95
	Cys145:H – Lig:O3	Hydrogen Bond	2.53
	Lig:H15 – Glu166:O	Hydrogen Bond	1.99
	Gln189:HE21 – Lig:O4	Hydrogen Bond	1.90
	His41 - Lig	Hydrophobic (Pi-Pi Stacking)	5.41
V120	Asn142:HD21 – Lig:O1	Hydrogen Bond	2.20
	Gly143:H – Lig:O1	Hydrogen Bond	2.04
	Cys145:HG – Lig:N4	Hydrogen Bond	2.50
	Lig:H9 – Glu166:O	Hydrogen Bond	2.09
	Lig:H14 – Glu166:O	Hydrogen Bond	1.97
	Gln192:HE21 – Lig:O2	Hydrogen Bond	2.00
	His41 - Lig	Hydrophobic (Pi-Pi Stacking)	4.21
V245	Thr25:HG1 – Lig:O1	Hydrogen Bond	1.95
	Thr26:H – Lig:O1	Hydrogen Bond	1.93
	Gly143:H – Lig:N2	Hydrogen Bond	1.99
	Cys145:H – Lig:N2	Hydrogen Bond	3.24
	Glu166:H – Lig:O3	Hydrogen Bond	3.28
	His41 - Lig	Hydrophobic (Pi-Pi Stacking)	5.12
V226	His41:HD1 – Lig:O1	Hydrogen Bond	1.79
	Asn142:HD21 – Lig:N2	Hydrogen Bond	2.20
	Lig:H25 – Glu166:O	Hydrogen Bond	2.25
	Gln189:HE21 – Lig:O3	Hydrogen Bond	2.07
	His41 - Lig	Hydrophobic (Pi-Pi Stacking)	5.26
V252	Lig:H17 - Leu141:O	Hydrogen Bond	2.17
	Asn142:HD21 - Lig:O1	Hydrogen Bond	2.08
	Gly143:H - Lig:O1	Hydrogen Bond	2.07
	Gly143:H - Lig:O2	Hydrogen Bond	2.33
	Cys145:H - Lig:O2	Hydrogen Bond	2.40
	Lig:H9 - His164:O	Hydrogen Bond	1.99
	Gln189:HE21 - Lig:O3	Hydrogen Bond	2.85
	Gln192:H - Lig:O4	Hydrogen Bond	1.95
V154	Thr26:H – Lig:O2	Hydrogen Bond	2.25
	Gly143:H – Lig:O1	Hydrogen Bond	1.74
	Cys145:H – Lig:O1	Hydrogen Bond	2.99

	Gln192:H – Lig:Br1	Halogen Bond	2.34
	His41 – Lig	Hydrophobic (Pi-Pi Stacking)	4.28
V304	Lig:N1 - Glu166:OE2	Salt bridge	4.49
	Lig:H7 – Thr25:OG1	Hydrogen Bond	2.19
	Ser144:HG – Lig:N3	Hydrogen Bond	2.53
	Ser144:H – Lig:N4	Hydrogen Bond	2.93
	Cys145:H – Lig:N4	Hydrogen Bond	2.89
	Glu166:H – Lig:O1	Hydrogen Bond	1.83
	Lig:H2 – Glu166:OE1	Hydrogen Bond	2.01
	His41 – Lig	Hydrophobic (Pi-Pi Stacking)	4.98
	His41 – Lig	Hydrophobic (Pi-Pi Stacking)	5.17
	His41 – Lig	Hydrophobic (Pi-Pi Stacking)	5.37
V172	Thr26:H – Lig:O2	Hydrogen Bond	1.90
	Lig:H4 – Gln189:OE1	Hydrogen Bond	1.85
	Gly143:H – Lig:O1	Hydrogen Bond	2.07
	Ser144:H – Lig:O1	Hydrogen Bond	2.32
	Cys145:H – Lig:O1	Hydrogen Bond	2.34
V97	His41:HD1 – Lig:O2	Hydrogen Bond	1.84
	Thr26:H – Lig:O3	Hydrogen Bond	2.06
	Glu166:H – Lig:N2	Hydrogen Bond	2.15
	Lig:H1 – Glu166:O	Hydrogen Bond	2.32
	Gln189:HE21 – Lig:O1	Hydrogen Bond	1.84
	His41 – Lig	Hydrophobic (Pi-Pi Stacking)	4.92
V111	Thr26:H – Lig:O2	Hydrogen Bond	2.07
	Lig:H6 – His41:NE2	Hydrogen Bond	2.58
	Asn142:HD21 – Lig:O1	Hydrogen Bond	2.11
	Gly143:H – Lig:O1	Hydrogen Bond	1.91
	Lig:H2 – His164:O	Hydrogen Bond	2.86
	Lig:H3 – Val186:O	Hydrogen Bond	3.44
	Arg188:O – Lig:H3	Hydrogen Bond	2.10
	Ser46:H – Lig:Br1	Halogen Bond	2.71
V238	His41:HD1 – Lig:O2	Hydrogen Bond	2.02
	Asn142:HD21 – Lig:O3	Hydrogen Bond	1.98
	Gly143:H – Lig:O3	Hydrogen Bond	2.26
	Gln192:HE21 – Lig:N2	Hydrogen Bond	3.15
	His41 – Lig	Hydrophobic (Pi-Pi Stacking)	4.73
V60	Thr26:H – Lig:O3	Hydrogen Bond	2.15
	Gly143:H – Lig:O2	Hydrogen Bond	2.00
	Cys145:H – Lig:O2	Hydrogen Bond	2.96
	His41:HD1 – Lig:N3	Hydrogen Bond	1.96
	Lig:H6 – Arg188:O	Hydrogen Bond	2.76
	His41 – Lig	Hydrophobic (Pi-Pi Stacking)	4.17
V103	Thr24:O – Lig:H8	Hydrogen Bond	2.19
	Ser46:H – Lig:N2	Hydrogen Bond	2.37
	Gly143:H – Lig:O2	Hydrogen Bond	1.74
	Cys145:H – Lig:O2	Hydrogen Bond	3.37
	Glu166:H – Lig:O3	Hydrogen Bond	1.85
Nilotinib	Asn142:HD22 - Lig:O1	Hydrogen Bond	2.56
	Asn142:HD21 - Lig:N6	Hydrogen Bond	2.33
	Cys145:HG - Lig:N5	Hydrogen Bond	2.48
	Lig:H16 - His164:O	Hydrogen Bond	2.58
	Lig:H9 - Glu166:OE2	Hydrogen Bond	1.96
	Gln189:HE21 – Lig:N6	Hydrogen Bond	2.01
	Gln189:HE22 - Lig:O1	Hydrogen Bond	2.06

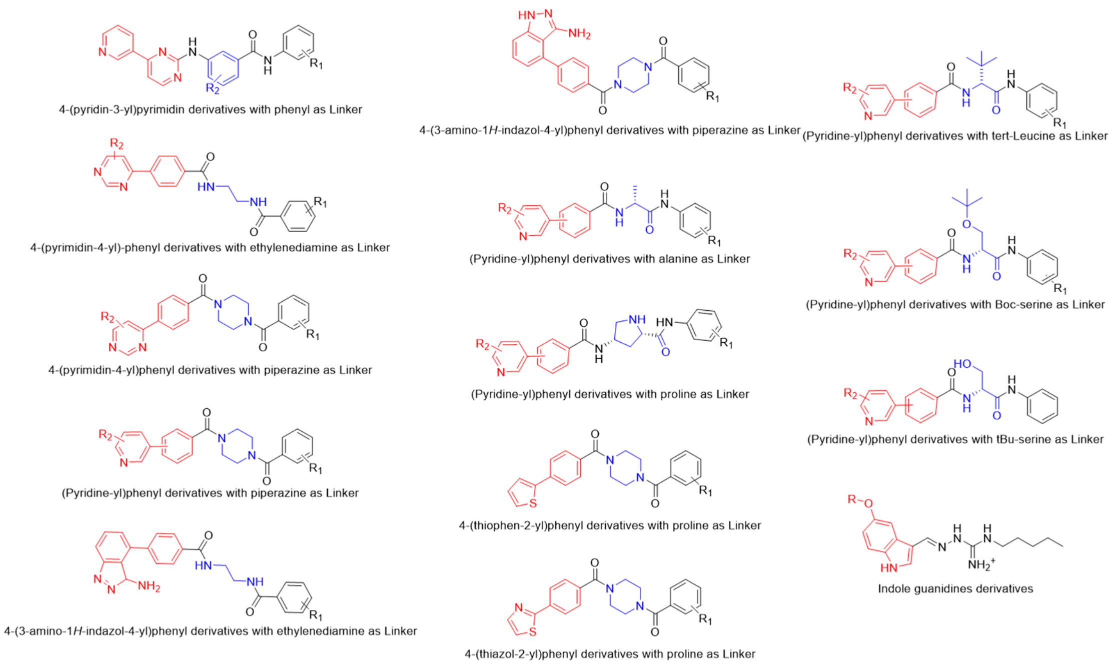

Figure S1. Structure types of all compounds.

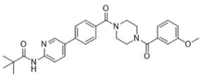
V75
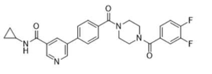
V97
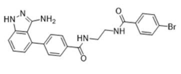
V111
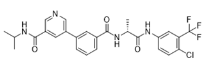
V131
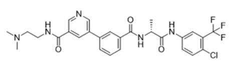
V133
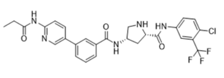
V139
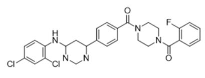
V159
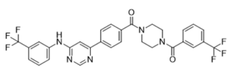
V172
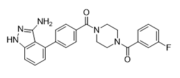
V205
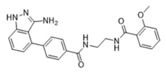
V222
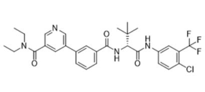
V226
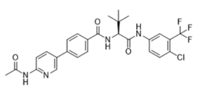
V231
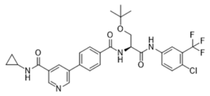
V243
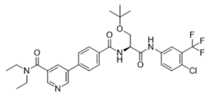
V245
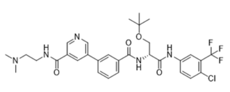
V247
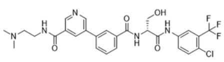
V253
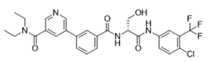
V254
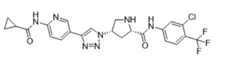
V282
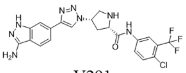
V291
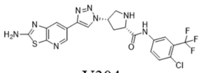
V304

Figure S2. Structures of the specific 20 compounds selected for MD analyses.

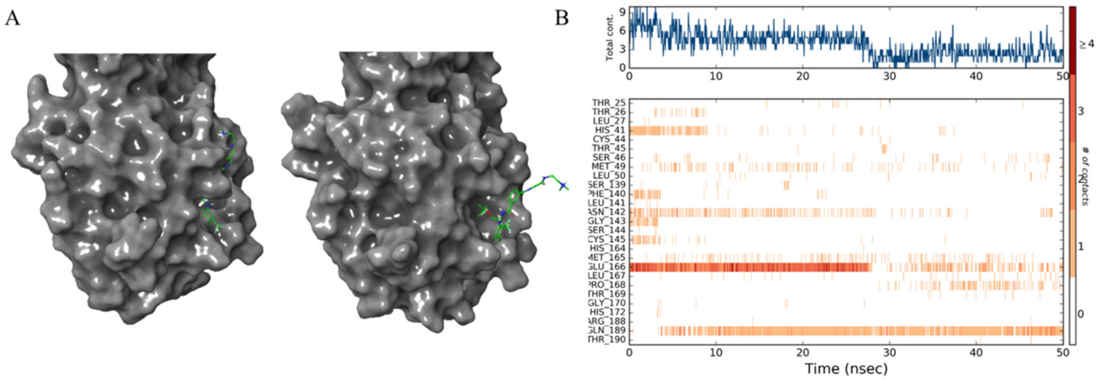

Figure S3. (A) Spatial structure of V247-M^{pro} complex at different times during dynamic simulation. At 1ns, the molecule binds closely to the protein (left); at 28ns, N-(2-dimethylamino)ethyl)nicotinamide of ligand structure has separated from the protein surface (right). (B) The interaction between V247 and the residues of the M^{pro} during 20 ns. Top panel shows the total number of specific contacts the protein makes with the ligand over the course of the trajectory, and bottom panel shows which residues interact with the ligand in each trajectory frame.

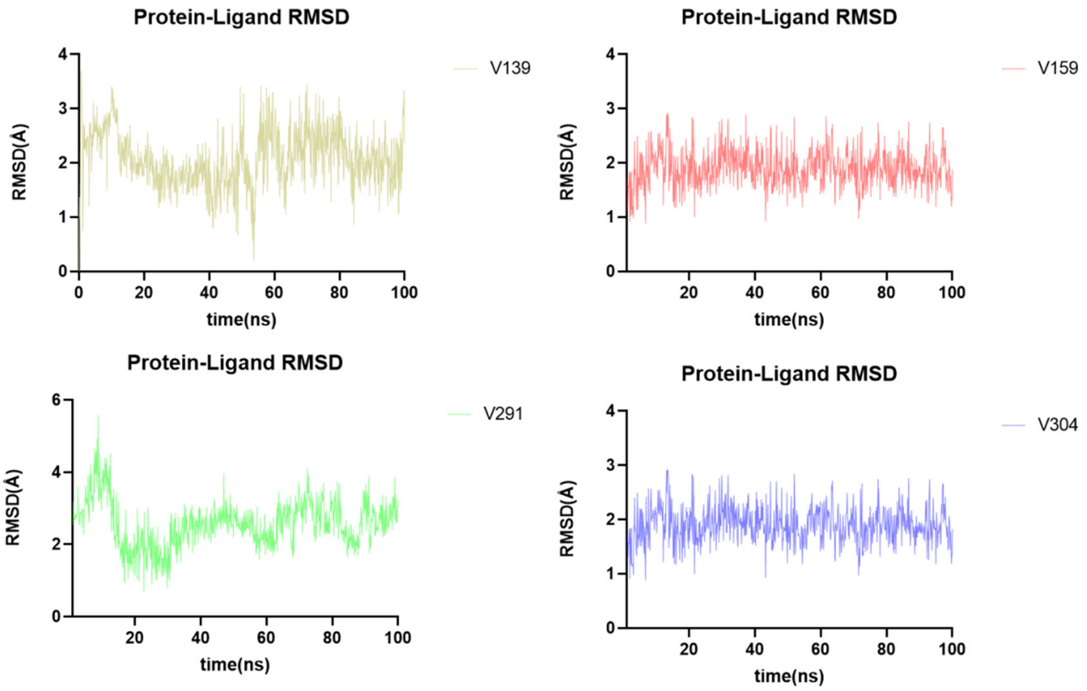

Figure S4. Results of 100ns molecular dynamics simulations of 4 representative compound-protein complexes.

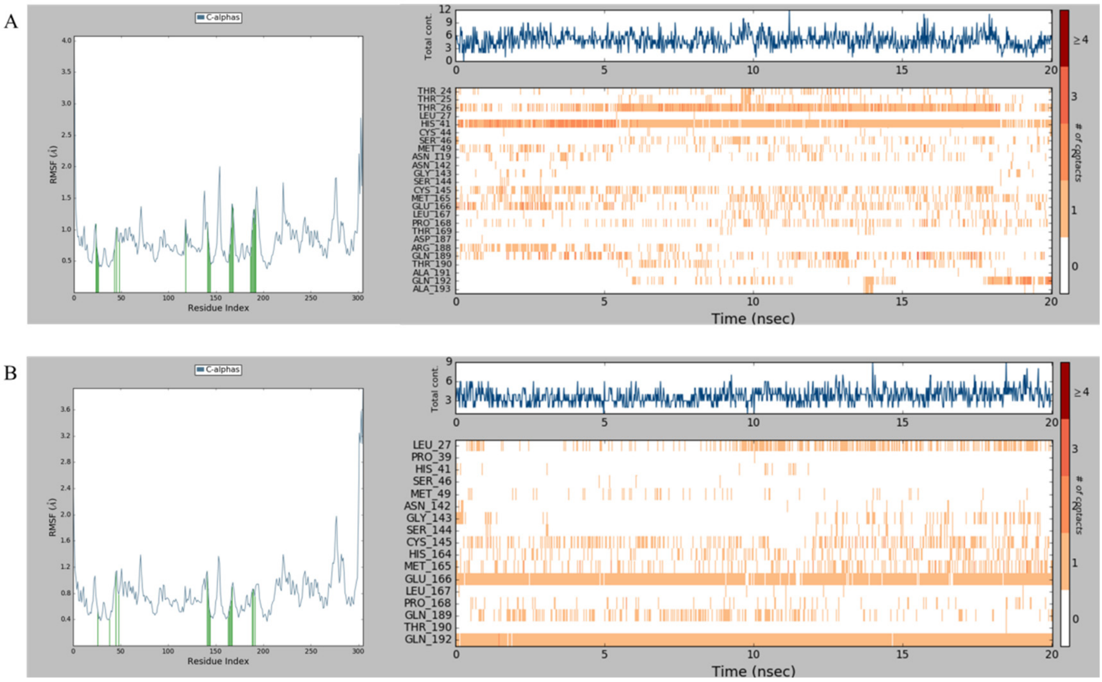

Figure S5. Protein RMSF plot (left) and Protein-Ligand Contacts (right) of V75-M^{pro} complex (A) and V97-M^{pro} complex (B)

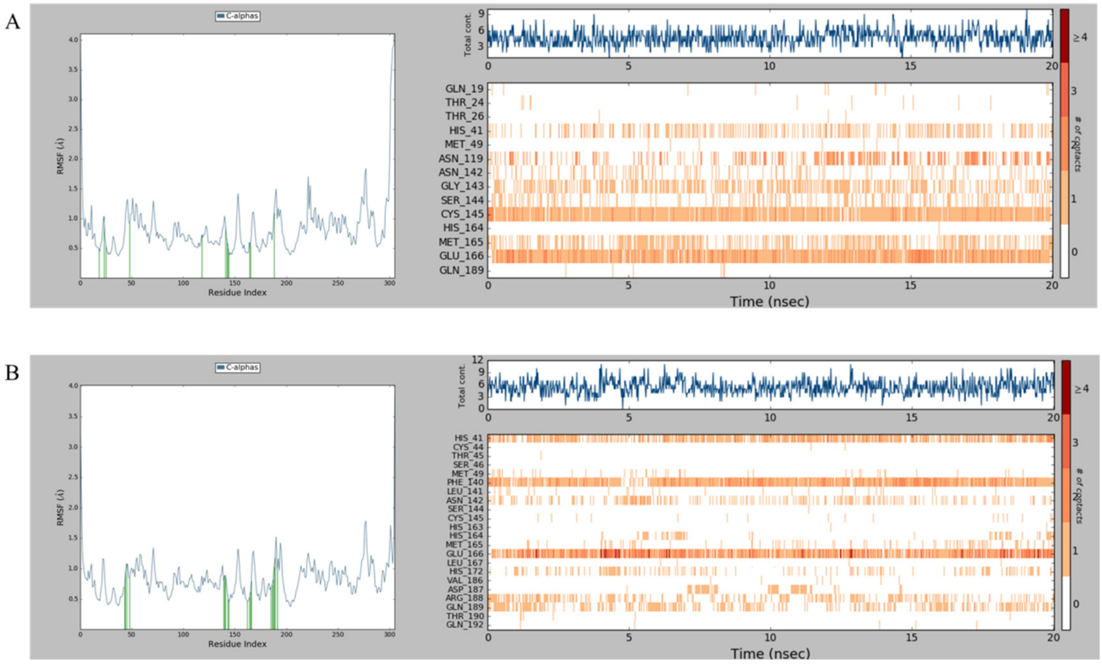

Figure S6. Protein RMSF plot (left) and Protein-Ligand Contacts (right) of V111-M^{pro} complex (A) and V139-M^{pro} complex (B)

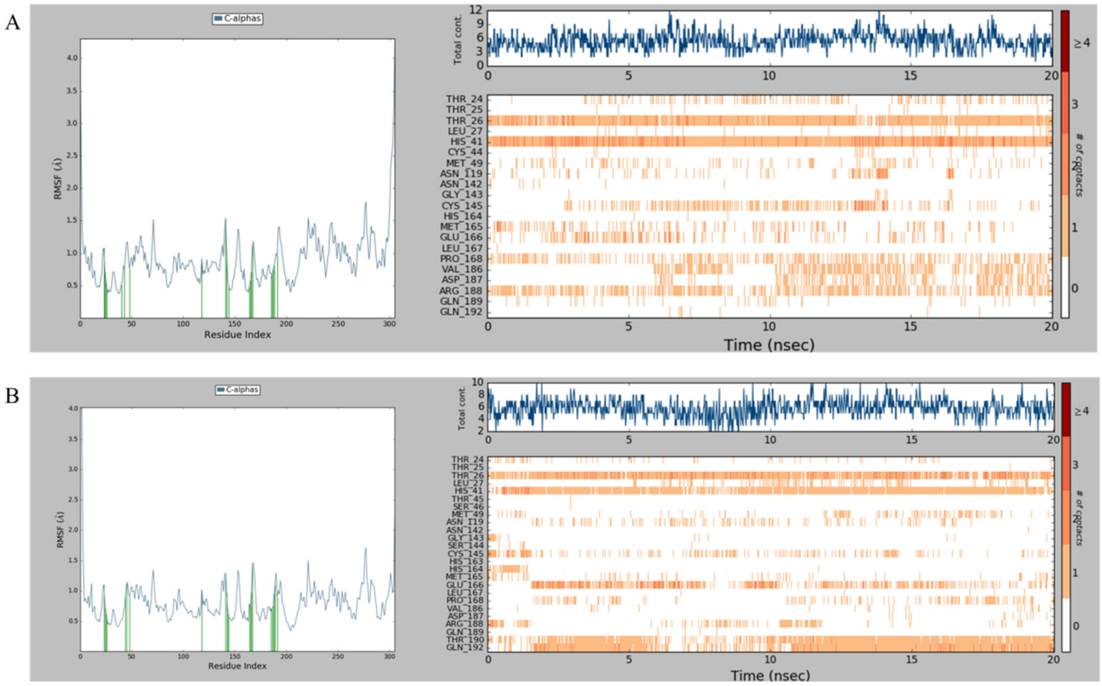

Figure S7 Protein RMSF plot (left) and Protein-Ligand Contacts (right) of V159-M^{pro} complex (A) and V205-M^{pro} complex (B)

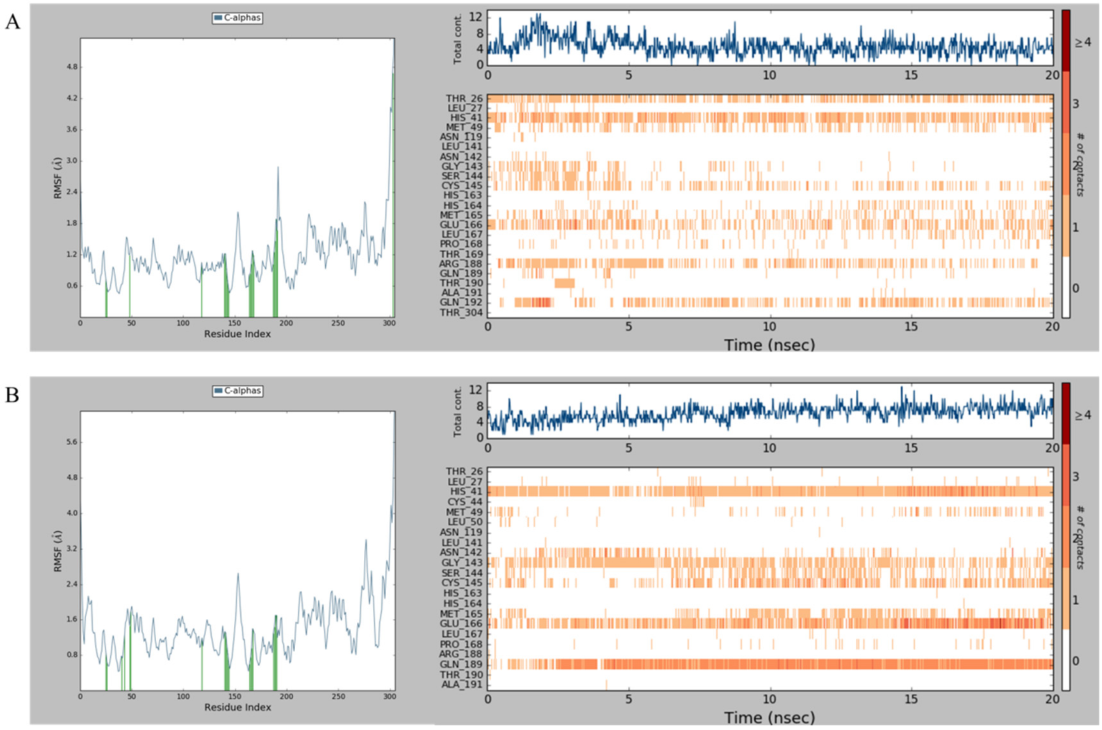

Figure S8. Protein RMSF plot (left) and Protein-Ligand Contacts (right) of V222-M^{pro} complex (A) and V226-M^{pro} complex (B)

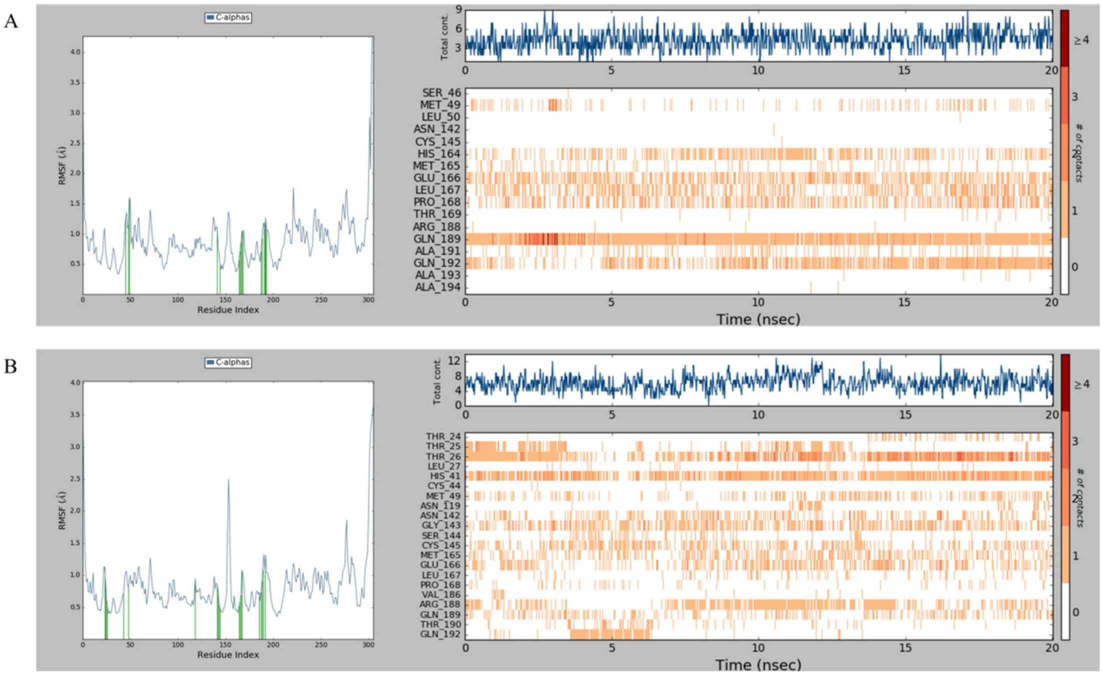

Figure S9. Protein RMSF plot (left) and Protein-Ligand Contacts (right) of V231-M^{pro} complex (A) and V243-M^{pro} complex (B)

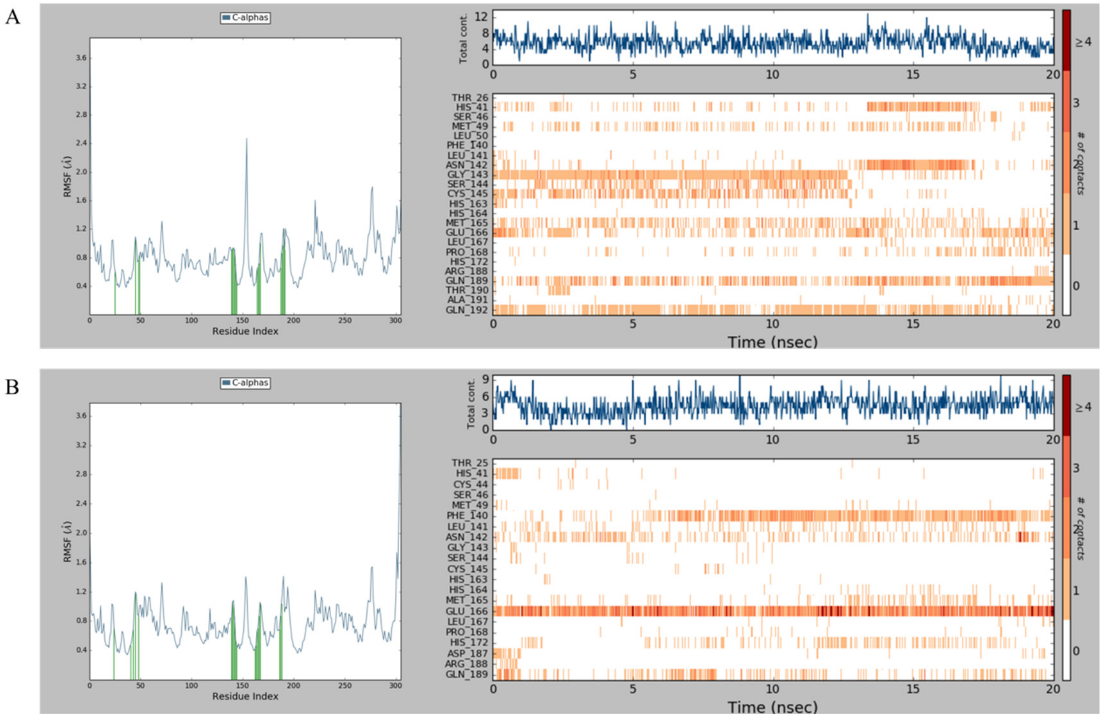

Figure S10. Protein RMSF plot (left) and Protein-Ligand Contacts (right) of V254-M^{pro} complex (A) and V282-M^{pro} complex (B)

A

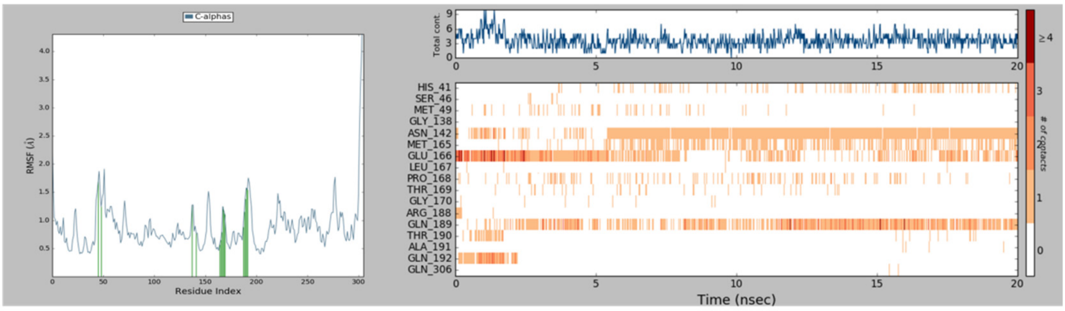

B

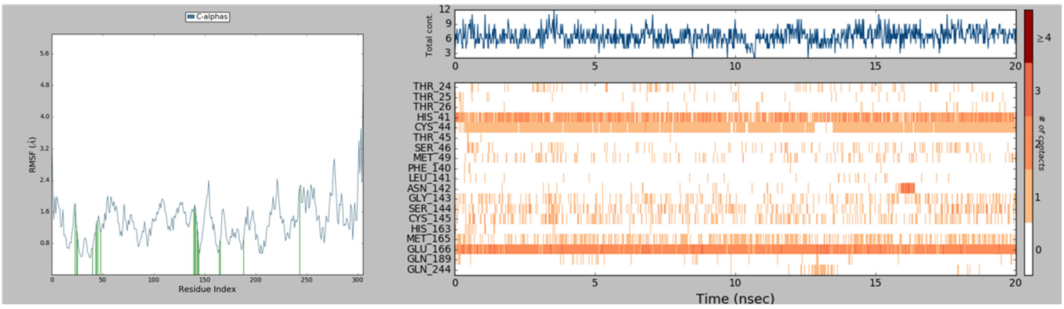

Figure S11. Protein RMSF plot (left) and Protein-Ligand Contacts (right) of V291-M^{pro} complex (A) and V304-M^{pro} complex (B)
